# Costello Syndrome and Umbilical Ligament Rhabdomyosarcoma in Two Pediatric Patients: Case Reports and Review of the Literature

**DOI:** 10.1155/2017/1587610

**Published:** 2017-01-19

**Authors:** Carlos Sánchez-Montenegro, Alejandra Vilanova-Sánchez, Saturnino Barrena-Delfa, Jair Tenorio, Fernando Santos-Simarro, Sixto García-Miñaur, Pablo Lapunzina, Leopoldo Martínez-Martínez

**Affiliations:** ^1^Pediatric Surgical Oncology Section, Oncology Service, Department of Hematology-Oncology, Hospital Nacional de Niños “Dr. Carlos Sáenz Herrera”, Paseo Colón, 1654-1000 San José, Costa Rica; ^2^Oncology Section, Department of Pediatric Surgery, Hospital Universitario La Paz, Paseo de la Castellana 261, 28046 Madrid, Spain; ^3^Institute of Medical and Molecular Genetics (INGEMM), Instituto de Investigación Hospital Universitario La Paz (IdiPAZ), Paseo de la Castellana 261, 28046 Madrid, Spain; ^4^Centro de Investigación Biomédica en Red de Enfermedades Raras (CIBERER), Instituto de Salud Carlos III (ISCIII), Calle Sinesio Delgado 4, 28029 Madrid, Spain

## Abstract

Costello syndrome is caused by heterozygous de novo missense mutations in the protooncogene* HRAS *with tumor predisposition, especially rhabdomyosarcoma. We here report two pediatric patients with Costello syndrome and umbilical ligament rhabdomyosarcoma. A review of the literature published in English in MEDLINE from January 1971 to June 2016 using the search terms “Costello syndrome” and “rhabdomyosarcoma” was performed, including two new cases that we describe. Twenty-six patients with Costello syndrome and rhabdomyosarcoma were recorded with mean age of diagnosis of 2 years and 8 months. The most common tumor location was the abdomen/pelvis, including four out of ten of those in the umbilical ligament. The most common histological subtype was embryonal rhabdomyosarcoma. Overall survival was 43%. A total of 17 rhabdomyosarcomas in pediatric patients arising in the umbilical ligament were recorded with mean age of diagnosis of 3 years and 4 months. Overall survival was 69%. Costello syndrome is a poorly known disorder in pediatric oncology but their predisposition to malignancies implies the need for a new perspective on early diagnosis and aggressive medical and surgical treatment.

## 1. Introduction

Costello syndrome (CS) (OMIM #218040) is a rare disorder with a distinctive prenatal presentation, postnatal feeding difficulties and failure to thrive, characteristic facial appearance (coarse features, full lips, and large mouth), abnormalities of the heart (hypertrophic cardiomyopathy, pulmonary valve stenosis, and tachyarrhythmia), skin and musculoskeletal system (soft skin, deep palmar and plantar creases, papillomata, sparse or curly hair, joint laxity, ulnar deviation of wrist and fingers, and tight Achilles tendons), and tumor predisposition such as rhabdomyosarcoma (RMS), neuroblastoma, or transitional cell carcinoma of the bladder [1]. It was first described in 1971 by Costello, a New Zealand pediatrician, based on its distinctive phenotype [2–4]. CS is caused by heterozygous de novo missense mutations in the protooncogene Harvey rat sarcoma viral oncogene homolog* (HRAS)* (chromosome 11p15.5). These mutations are present in 100% of patients with CS, but they are usually confirmed in 80–90% due to an inaccurate clinical diagnosis [4, 5]. Mutations result in a gain-of-function of the abnormal protein product and increased activation of the Mitogen activated protein kinase (MAPK) pathway [1]. The most common tumor associated with CS is RMS, mainly involving abdomen, pelvis, and/or urogenital area [6]. The first two CS patients associated with RMS were described in 1998 [7]; before that date this syndrome was mainly associated with benign tumors, especially papillomata [3].

We here report two children with CS and RMS of the umbilical ligament (UL). No prior correlation of this tumor location with CS had been described so far.

## 2. Case Presentation

### 2.1. Case  1

**Figure 1:**
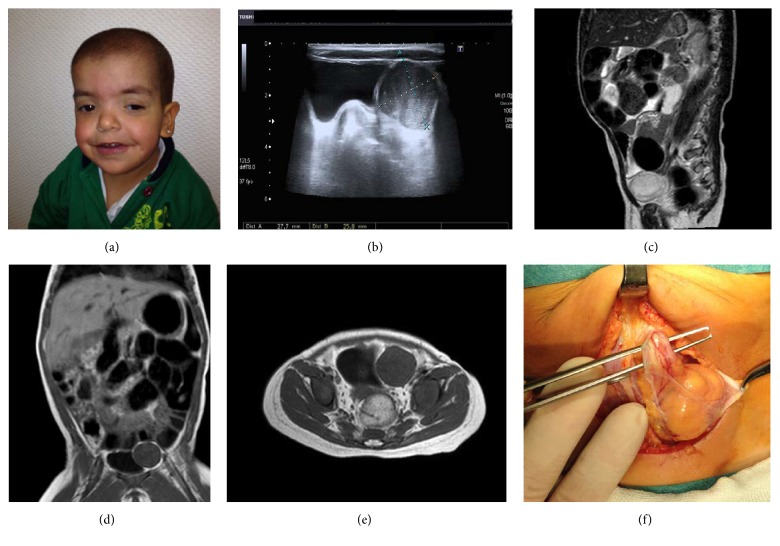
Patient 1. (a) Photograph of the physical features of the patient with low-set ears, lower palpebral fissures, hypertelorism, broad nasal bridge, thick lips, and thin hair. (b) Abdominal ultrasound showing the presence of a solid mass paravesical displacing the left ovary and bladder without infiltrate surrounding structures. (c) Initial MRI showing a hyperintense tumor predominantly heterogeneous intensity on T2-weighted sequence compressing the bladder. ((d) and (e)) MRI on T1-weighted sequences after 3 cycles of chemotherapy with slight decrease in left paravesical oval mass. (f) Operative image indicating the presence of tumor originating in the left medial UL.

This is a 4.4-year-old female. She is third child of healthy unrelated parents with no family history of note. Pregnancy was complicated with polyhydramnios. The patient was born from a preterm delivery (33 weeks), large (2840 gr) for gestational age. She presented feeding difficulties from the beginning that required nasogastric tube feeding and later gastrostomy, at the age of 4 months. Cardiological examination showed mild pulmonary stenosis. She was referred to the Genetics Clinic for assessment at the age of 6 months because of failure to thrive, hypotonia, and dysmorphic features. On examination she had features highly suggestive of a RASopathy, but on account of the skin findings (sparse hair, glabellar capillary malformation, a large 4 × 12 cm café-au-lait spot in her right flank, and two blue nevi in right temporal region and left groin), difficult to differentiate with certainty between CS and Cardiofaciocutaneous syndrome. However,* HRAS* mutation analysis demonstrated a common recurrent mutation (p.G12S), confirming the diagnosis of CS in this child at the age of 7 months (Figure 1(a)). Because of the high risk of tumor development associated with this condition, close follow-up was indicated according to the recommended tumor screening protocol [8, 20]. An abdominal ultrasound scan performed at the age of 16 months revealed a 31 × 32 mm paravesical solid pelvic mass, with minimal intralesional vascularity; no intraabdominal free fluid or lymphadenopathies were seen. The ovaries were not involved (Figure 1(b)). Magnetic resonance imaging (MRI) showed a left paravesical tumor (3 × 5 × 2.7 cm) without associated lymphadenopathies, hypointense on T1-weighted sequences, and hyperintense with predominantly heterogeneous intensity on T2-weighted sequences. The mass caused compression and displacement of the bladder to the right side (Figure 1(c)). Tumor markers were as follows: beta-subunit of human chorionic gonadotropin (*β*-hCG) < 2 mUI/mL (normal < 6.0), alpha-fetoprotein (*α*FP) 2.9 ng/mL (normal < 8.0), carcinoembryonic antigen (CEA) 0.8 ng/mL (normal < 5.0), CA-15.3 19.7 UI/mL (normal < 32.0), CA-19.9 43.7 UI/mL (normal < 37.0), and neuron specific enolase (NSE) 27.86 ng/mL (normal 0–16.0). Thoracic computed tomography (CT) and scintigraphy showed no evidence of metastasis. Bone marrow biopsy was normal. Tru-Cut biopsy guided by ultrasound reported an embryonal RMS with a proliferation index Ki-67 of 60%, mesenchymal malignant round cell, and short spindle tumor: vimentin (+), desmin (focal +), MyoD1 (focal +), CD99 (+ focal staining), S100 protein (−), pancytokeratins AE1/AE3 (−), synaptophysin (−), and CD99 (−). She was enrolled on the standard risk group protocol European pediatric Soft tissue Sarcoma Study Group (EpSSG) RMS 2005 subgroup D with 3 neoadjuvant chemotherapy cycles. Preoperative MRI showed a slight decrease in tumor size (3.2 × 3 × 2.5 cms) (Figures 1(d) and 1(e)). At the definitive surgery a 5 cm tumor dependent of the remaining left umbilical artery (left medial UL), located slightly to the left and displacing the bladder dome, was found (Figure 1(f)). Complete resection of the tumor was performed. No pathological lymph nodes were observed. Final pathology report showed an embryonal RMS with signs of response to chemotherapy (maturation phenomenon at cytoplasmic and nuclear level of the tumor cellularity), with maximum cell proliferation index Ki-67 of 5–10%, almost all undifferentiated except in a small region; resection margins were negative for tumor (1–4 mm from the edges of radial resection margin and 3 cm of UL). Chemotherapy was then continued until completing 25 weeks and coadjuvant radiotherapy was administrated after the completion of chemotherapy. At 23 months of follow-up after diagnosis, no residual tumor or relapse has been identified.

### 2.2. Case 2

**Figure 2:**
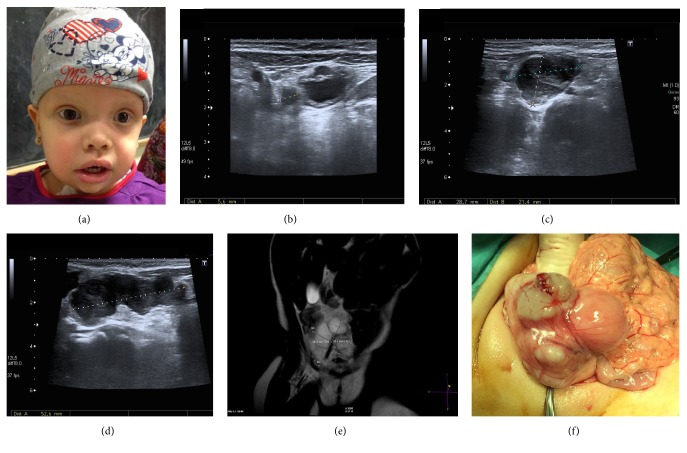
Patient 2. (a) Photograph of the physical features of the patient with prominent forehead, low-set ears, lower palpebral fissures, hypertelorism, broad nasal bridge, thick lips, and anteverted slightly nostrils. (b) Initial abdominal ultrasound showing the presence of a tubular image caliber of 5.6 mm extending in the theoretical location of the right umbilical artery. ((c) and (d)) Follow-up abdominal ultrasound in the midline showing a multilobulated hypoechoic solid tumor with marked hypervascularization, extending from the proximity of the abdominal wall to the right supravesical region. (e) MRI onT2-weighted sequence confirms the presence of several nodular masses grouped with thickening of both medial umbilical folds, the right to 4 mm proximal to the umbilicus and 8 mm adjacent to the iliac vessels paravesical region portion. (f) Operative image indicating the presence of tumor originating in the right medial UL.

This is a 5.4-year-old female. She is the first and only child of a healthy nonconsanguineous couple with unremarkable family history. Pregnancy was complicated with polyhydramnios and delivery was preterm (34 weeks), with a high birth weight (2980 g, +2.6 SD). She was admitted to the neonatal ward for investigation of a suspected dysmorphic syndrome and initial* FGFR3* molecular genetic testing for achondroplasia revealed no mutations. Feeding difficulties and failure to thrive were noted from the beginning requiring nutritional intervention and gastrostomy feeding from the age of 10 months. She had a heart murmur with a normal echocardiogram. She also had bronchomalacia and at MRI nonspecific white matter hyperintensities in CNS. She was referred to our clinical Genetics Clinic for evaluation at the age of 4 months and in view of her previous history and clinical findings CS was suspected. HRAS Sanger sequencing detected the common c.34G>A (p.G12S) mutation confirming the diagnosis of CS (Figure 2(a)). As part of the follow-up the recommended tumor screening protocol was arranged and at the age of 3 years she underwent an abdominal ultrasound that showed a tubular image running through the theoretical place of the umbilical arteries, with a larger caliber on the right side of 5.6 mm, and associated echoes inside without Doppler flow (Figure 2(b)). Two subsequent ultrasounds did not show any abnormality. A new abdominal ultrasound 9 months later reported the presence of a hypoechoic multilobulated solid tumor (5.3 × 2.8 cm) in the midline extending to the right in supravesical region with marked hypervascularization and extending cranially to the vicinity of the abdominal wall (Figures 2(c) and 2(d)). MRI showed a solid abdominal tumor (6 × 5.5 × 3 cm) composed of nodular masses grouped in the UL region with involvement of the medial umbilical folds that were thickened throughout the iliac vessels region (Figure 2(e)). No regional metastatic disease was observed. Tumor markers were as follows: *α*FP < 1.3 ng/mL, CEA 5.3 ng/mL, and CA-19.9 UI/mL. No lung metastases were observed in the thoracic CT scan and bone marrow biopsy was normal. She underwent a complete tumor resection (Figure 2(f)). The mass was located on the right medial UL (from its insertion into the right iliac artery to the umbilicus). Pathology report showed a spindle cell RMS with microscopic positive margins in 2 submitted samples (8.5 × 5 × 3 cm and 3.5 × 1.5 × 1 cm) and one resected lymph node was negative for malignancy. Tumor was composed of spindle cells that form cross linked bundles varying degrees, with elongated oval nuclei, vesicular configurations with coarse chromatin. The tumor had a pattern of nodular growth, markedly myxoid stroma with microcystic focal degeneration and 5% tumor necrosis; no lymphovascular invasion, maximum proliferation index Ki67 of 30%; muscle specific actin (+), desmin (+), WT1 (+ cytoplasmic), MyoD1 (+ nuclear), and CD99 (−). Two weeks after surgery, the standard risk group protocol EpSSG RMS 2005 subgroup B (Stage I) chemotherapy was started that includes ifosfamide, vincristine, and actinomycin D. At 18 months of follow-up after diagnosis, no residual tumor or relapse has been identified.

## 3. Methods

We describe two new cases of RMS and CS, based on medical records of patients diagnosed at the Institute of Medical and Molecular Genetics and treated at the Departments of Pediatric Hematology-Oncology and Pediatric Surgery, University Hospital La Paz (Madrid, Spain). A review of the literature published in English in MEDLINE from January 1971 to June 2016 using the search terms CS and RMS was done; also the references contained in these articles even if they were not indexed in MEDLINE were analyzed. A review from January 1933 until June 2016 of all reported pediatric cases of UL RMS, including as search criteria UL, RMS, urachus, and umbilical arteries, was performed.

## 4. Results

In all cases analyzed and reviewed (same cases reported in more than one publication were counted once), it was possible to document the presence of 26 patients with CS and RMS (Table 1) [5–25], including the 2 cases described herein. The mean age of diagnosis was 2 years and 8 months (range: 6 months–7 years). It was slightly more common in females than in males (1.2 : 1). The commonest location was the abdomen/pelvis (10/20), including 4 out of 10 of those in the UL. The most common histological type was embryonal RMS (14/19). Seven out of nine patients who died (and with reported histology) showed embryonal RMS. The most frequent encountered* HRAS *mutation associated with RMS was p.G12S (7/12). Only 4 deaths were documented in patients with change in codon 12 (2 patients G12S, 1 patient G12A, and 1 patient G12C). Chemotherapy that was applied varies based on the protocols of the countries where they were diagnosed (Table 2) [5–10, 12–14, 16–25]. Overall survival was about 43% (9/21). Mortality was observed in 12 out of 21 individuals; in nine of them associated with neoplasia; and in three associated with surgery.

Similarly, the total number of reported cases of UL RMS in pediatric patients, either in the obliterated umbilical arteries (medial UL) or in urachus (median UL), is 17 including these 2 new cases (Table 3) [5, 7–9, 24–31]. The mean age of diagnosis was 3 years and 4 months (range: 4 months–6 years). It is slightly more common in females than in males (1.4 : 1). The most common location was the urachus (15/17). The most common histological type was embryonal RMS (12/15) and one-fourth (3/12) of patients who died with reported histology showed embryonal RMS. Chemotherapy varies based on the protocols of the countries where they were diagnosed (Table 4) [7, 24, 26–31]. Overall survival was 69% (11/16), overall mortality was 31% (5/16), two-year absolute survival rate was 69% (11/16), and five-year absolute survival rate was 19% (3/16).

## 5. Discussion

The prevalence of CS is estimated to be 1 : 1,290,000 individuals in Japan and at least 1 : 500,000 in the United Kingdom; the incidence was estimated to be 1 : 60,000–100,000 [4, 22]. The overall tumor incidence is approximately 10–15% over the lifetime of individuals with an identified* HRAS* mutation [1, 4, 9], RMS accounting in about 60% of all neoplasia [1]. Based on the 300 cases of CS currently known [4], we found an incidence of 8.7% RMS in patients with CS.

CS is the first disorder associated with germline mutations in the Ras family of guanosine-5′-triphosphate (GTP)ases [21].* HRAS *has six exons; five exons are encoded for a protein of 189 amino acids with a molecular weight of 21 kDa [4]. Missense mutations at codons 12 and 13 are in constitutively active GTP-bound conformation and activate downstream effectors such as MAPK, phosphatidylinositol 3-kinases (PI3K), and ral guanine nucleotide dissociation stimulator (RalGDS) [16, 21], in signaling pathways controlling cell proliferation and differentiation [9].

The majority of patients with CS had 34G>A transition in codon 12 (80%) [4, 16]. More than 95% of the mutations are predicted to result in substitution of the glycine in position 12 or 13 of the protein product [1]. All individuals with malignancy had a codon 12 mutation (especially G12A, 4/7 patients) [5, 21]. The rare G12V mutation is associated with a more severe, early lethal phenotype; some patients die from respiratory distress, hypertrophic cardiomyopathy, or malignant tachycardia prior to being diagnosed with CS [1]. As far as we know, no individual with G13C has developed a malignant tumor [4, 32].

RMS is the most common soft tissue sarcoma of infancy. Its incidence is between 4 and 7 cases per 1 million children younger than 15 years [8, 12]. The age peak is between 2 and 5 years with a slight male predominance (1.2–1.4/1). These tumors may arise anywhere in the body and fewer than 20% are located in the pelvis [12]. Most RMS tumors are originated in the head and neck region, urogenital tract, and extremities [33]. The most common histologic type seen in >50% is embryonal, followed by alveolar in 20–30%. Pleomorphic RMS is rare (1%) and has a poor prognosis [8]. Alveolar RMS is characterized by the translocation t(2;13)(q35;q14) in 70% of cases and the variant translocation t(1;13)(p36;q14) in a smaller percentage of cases. Evidence accumulates that alveolar RMS and embryonal RMS are two different disorders: while alveolar RMS may originate from primitive uncommitted mesodermal cells, embryonal RMS originates probably from more differentiated myoblasts. Ras protein-specific guanine nucleotide-releasing factor 1 (RasGRF1) plays an important role in alveolar RMS pathogenesis. These interesting concepts however need more evidence [33].

The umbilicus caudally originates the common ligament, which is divided into obliterated umbilical arteries (medial UL) and urachus (median UL). The urachus varies from 3 to 10 cm in length and from 8 to 10 mm in diameter. It is a three-layered tubular structure, the innermost layer being lined with transitional epithelium in 70% of cases and with columnar epithelium in 30%. The structure is surrounded by connective tissue and outermost muscular layer in continuity with the detrusor muscle. Along its path from the bladder to the umbilicus, the urachus lies between the transverse fascia and the parietal peritoneum contained in the pyramidal, retropubic, perivesical preperitoneal space compartmentalized by umbilicovesical fascia, along with the medial umbilical ligaments and the bladder. Occasionally, the urachus may merge with one or both of the obliterated umbilical arteries (medial UL), and there may be a slight deviation to the right or left of the midline [34]. The urachus is an embryological remnant resulting from the obliteration of the allantoic channel, which attaches the bladder dome to the umbilicus [31].

Urachal malignant tumors are extremely rare, representing less than 0.5% of all bladder cancers and 0.01% of all tumors. They may arise from any portion of the urachus and are most commonly found in adults and males (60–70%). Although sarcomas represent only 8%, they are the most frequent urachal neoplasms reported in patients younger than 20 years of age (67%) [27]. Poor prognosis is a common feature in adults and pediatric urachal malignancies with 50% to 60% of 5-year survival. In contrast, the 5-year event-free survival in pelvic RMS was reported to be about 51% [31].

This indolent and rather large urachal tumor was mostly revealed by an uncomplicated progressing mass without prodromic urological symptoms. The delay of diagnosis could be explained by a long asymptomatic progression of the tumor in a preperitoneal location, which allows an important local spreading before diagnosis. Even in patients without tumor rupture, peritoneal involvement appeared to be the key point in the prognosis of urachal RMS. Systematic cytology at diagnosis could be more accurate to evaluate peritoneal extension in order to avoid understaging and adjust adjuvant locoregional treatment [31].

Previously, only 12 patients with RMS arising from the urachus (median UL) in children without CS have been reported in the English literature (Table 3). By 2011, there were 29 reported cases of cancer in patients with CS: 19 RMS (mean age of 2.3 years; 9 embryonal, 1 alveolar, 1 mixed histology, 1 pleomorphic, 1 spindle cell type, and 6 unclassified), 5 neuroblastomas, 4 bladder cancer, and 1 fibrosarcoma [35].

Loss of heterozygosity for 11p15.5 in RMS from individuals with CS suggests that loss of the wild-type allele is the second hit in tumor development [6, 16, 36]. Whereas the* HRAS* mutation in a sporadic malignancy in the general population is present only within the tumor cells, in CS an identical mutation is present in every single cell of the individual, thus accounting for the tumor predisposition [1], implicating an alternate mechanism of rhabdomyosarcogenesis [16]. In addition, one patient with CS and uniparental trisomy due to triplication of the mutated paternal* HRAS* gene was also reported. RMS typically displays loss of heterozygosity of the 11p15.5 region because of paternal uniparental disomy, and approximately 28% of cases harbor mutations in genes of growth signaling pathways. In reports of uniparental disomy plus* HRAS* mutation, the latter was heterozygous and therefore must have occurred after the development of uniparental disomy [24]. The functional analysis also revealed that CS-causing mutant HRAS proteins caused oncogene-induced senescence in human fibroblasts [37]. The important role of PI3K-protein-kinase B (AKT) signaling in Ras-mediated transformation and tumorigenesis is highlighted by constitutive AKT activation in RMS cell lines and clinical samples. Thus, prolonged PI3K-AKT signaling might also be implicated in the development of soft tissue sarcoma in patients with CS [38].

Only four out of 11 patients were diagnosed with CS prior to the RMS diagnosis, in 5 patients CS was diagnosed after the RMS, and in the remaining 2 the order was unknown [20]. In 2003, a further three unpublished cases of RMS in children with CS were known through the International CS Support Group [18], and for that reason, there is possibly underdiagnosis of RMS in patients with CS.

A screening protocol was proposed consisting of ultrasound examination of the abdomen and pelvis every 3–6 months until age of 8–10 years for RMS and abdominal neuroblastoma, urine catecholamine metabolite analysis every 6–12 months until age of 5 years for neuroblastoma, and urinalysis for hematuria annually for bladder carcinoma after age of 10 years. The prior diagnosis of CS was the prerequisite for the implementation of a tumor screening protocol [8]. The recommendation based on the presumed fast doubling time of RMS was abdominal sonography screening for RMS that should be done in similar intervals of every four months until stronger evidence would suggest otherwise [39]. Screening for neuroblastoma is no longer recommended because many CS patients without identifiable tumor show elevated catecholamine metabolites in urine; it appears that in this patient group an elevation above the normal limit, defined as 2 standard deviations (SD) above the mean for age, is more likely to be a variant, rather than a sign of a neuroblastoma [1, 40]. A French monitoring protocol proposed the determination of catecholamines and their metabolites in urine every six months until the age of 5 years and ultrasound abdominopelvic every six months until the end of the puberty, seeking hematuria dipstick from 6 months to the age of 10 [41]. The protocol in our hospital for patients with CS includes an abdominal and pelvic ultrasound 3 times during the first year of life and in childhood every 6 months until age of 10 [42]. We believe that the best option is to perform a pelvic abdominal ultrasound every 3 months during the first eight years of life and then controls according to medical criteria. This is even more necessary in patients with possible UL RMS because diagnosis was usually done in advanced stages with a poor survival. It is possible that with close monitoring after an early diagnosis will detect initial stages of the tumor in previously not described locations.

Head and neck MRI scans can be performed less frequently. Ear examination and tympanometry every 4 to 6 months to detect middle ear effusion, which would raise suspicion for a nasopharyngeal mass, are also recommended [6].

The outcome of CS patients with RMS has been comparable to that for patients without the syndrome, with a 5-year survival rate of 65% to 70% [19]; but according to this review it could be worse than the general population. Neoplasia was noted at the cause of death in five (22%) patients with CS [4, 15], but in our study the mortality associated with RMS was 43%. Because the long-term survival in RMS is based on the histology of the tumor and the extent of disease at diagnosis, early diagnosis may alter the treatment regimen and improve prognosis. This could be particularly important in patients with CS, who often are medically fragile [20].

The Ras/MAPK pathway is an attractive target in the treatment of cancer utilizing small molecule therapeutics that specifically inhibits the pathway. Ras pathway agents, such as farnesyltransferase inhibitors (tipifarnib and lonafarnib) that prevent posttranslational modification of Ras, are being evaluated for cancer treatment and may be of therapeutic use for syndromes in this pathway, especially CS. In addition, B-Raf protooncogene serine/threonine kinase (BRAF) and mitogen/extracellular signal-regulated kinase (MEK) inhibitors offer the same potential in the possible treatment of CS. Since the Ras/MAPK pathway has targets for inhibition in cancer treatment, there are many small molecule therapeutics that are in development or undergoing clinical trials, with some already FDA approval [43].

## 6. Conclusions

CS is a syndrome poorly known in oncology, but their predisposition to malignancies including UL RMS implies the need for a new perspective on early diagnosis and aggressive medical and surgical treatment.

## Figures and Tables

**Table 1:** Characteristics of patients with Costello syndrome and rhabdomyosarcoma.

PT number	Author	Year	Country	Sex	Age at diagnosis (years/months)	Symptoms	Location	Histology	Mutation AAC (NS)	Therapy	Outcome
1	Kerr et al. [7, 8]	1998	England	F	2 y 4 m	Vomiting Constipation	Retroperitoneal: left psoas mayor muscle	Embryonal	G12S (34G>A)^*∗*^ [9]	S, ChT	Relapse, tumor-free age of 7 years
2	Kerr et al. [7, 8]	1998	England	F	3 y 2 m	Abdominal mass	Pelvis: urachus (median UL), adherent to the bladder	Embryonal	G12A (35G>C) [5]	S, ChT	Histology showed extending up to the resection margin Died aged 5 years [5, 9]
3	Feingold [8, 10]	1999	USA	M	6 m	Tumor	Right foot	Alveolar	NDF	S, ChT	Tumor-free age of 20 years [11]
4	Bisogno et al. [8, 9, 12]	1999	Italy	M	1 y 8 m	Constipation Abdominal pain	Lower abdomen, pelvis: abdominal wall^*∗*^	Embryonal	NDF	S, ChT	Only biopsy Died aged 2 years and 3 months
5	Sigaudy et al. [8, 13]	2000	France	M	6 y	NDF	Inguinal/scrotal	Embryonal	NDF	NDF	NDF
6	Gripp et al. [8, 14, 15]	2002	USA	M	5 y 6 m	Abdominal pain	Pelvis: pulmonary metastases was present	Embryonal	NDF	S, ChT	Died after recurrence [14]
7	Gripp et al. [8]	2002	USA	F	3 y 7 m	Abdominal pain Emesis	Upper abdomen: mass encasing the superior mesenteric an celiac arteries. Single pulmonary nodule	Pleomorphic	NDF	ChT	Died aged 4 years
8	Gripp et al. [8]	2002	USA	F	2 y 6 m	Abdominal mass	Pelvis: bladder	Embryonal	NDF	ChT, RT, S	Died aged 7 years for recurrency of RMS/fibrosarcoma [16]
9	Gripp et al. [8]	2002	USA	NDF	7 m	Perineal growth	Perineal	Embryonal	NDF	ChT, RT, S	Tumor-free age of 2 years
10	Gripp et al. [8]	2002	USA	NDF	1 y 11 m	Proptosis	Left orbit	Unknown	NDF	S, RT	Tumor-free age of 4 years
11	Kawame et al. [17]	2003	Japan	F	1 y 4 m	Abdominal mass	Pelvis	Spindle cell	NDF	S, ChT	Tumor-free age of 7 years
12	Kerr et al. [18]	2003	UK	M	2 y 2 m	Large pelvic mass	Retrovesicular mass by ultrasound	Embryonal	NDF	ChT	Died aged 2 years and 4 months
13	O'Neal et al. [19]	2004	USA	F	3 y	Right-sided cervicofacial mass	Parameningeal infratemporal fossa: right periparotid masticator space region	Alveolar	NDF	ChT, S, RT	Clinical group 3 T2b N0 stage 3 Died during surgery
14	Gripp [20]	2005	USA	F	3 y 2 m	Facial mass	Right infratemporal fossa	Mixed alveolar and embryonal	NDF	ChT, S	Died during surgery by ventricular tachycardia followed rapidly by loss of heart rhythm
15	Aoki et al. [21, 22]	2005	Japan	F	7 y 2 m	NDF	NDF	NDF	G12S (34G>A)	NDF	NDF
16	Gripp et al. [9]	2006	USA	M	2 y	NDF	NDF	NDF	G12S (34G>A)	NDF	Tumor-free age of 11 years^*∗*^
17	Kerr et al. [5]	2006	UK	NDF	7 m	NDF	Metastatic arising from the prostate	Embryonal [23]	G12C (34G>T)	NDF	Died aged 7 months
18	Kerr et al. [5]	2006	UK	NDF	NDF	NDF	NDF	NDF	G12S (34G>A)	NDF	Died aged 7 years
19	Kerr et al. [5]	2006	UK	NDF	NDF	NDF	NDF	NDF	G12A (35G>C)	NDF	NDF
20	Lo et al. [23]	2008	UK	M	2 y 3 m	Respiratory function deteriorated	Right lung	NDF	G12S (34G>A)	S	Died few weeks later surgery
21	Ahmadi and Harley [6]	2010	USA	M	2 y	Increased work of breathing	Nasopharyngeal	Embryonal	NDF	S, ChT, RT	Clinical group IIA (T1N0M0) Tumor-free age of 3 years
22	Abe et al. [22]	2012	Japan	NDF	NDF	NDF	NDF	NDF	NDF	NDF	Died
23	Menke et al. [24, 25]	2015	Germany	F	3 y	Abdominal mass	Lower anterior abdomen, above the urinary bladder^*∗*^ (urachus-median UL by location)	Embryonal	G12C (34G>T)	S, ChT	R1 resection (marginal tumor residue). Tumor-free age of 6 years
24	Kratz et al. [25]	2015	Germany	M	1 y	NDF	NDF	Embryonal	G12S (34G>A)	NDF	NDF
25	Sánchez-Montenegro et al. (this report)	2017	Spain	F	2 y 4 m	Asymptomatic	Left medial UL	Embryonal	G12A (35G>C)	ChT, S, RT	Tumor-free age of 4 years and 4 months
26	Sánchez-Montenegro et al. (this report)	2017	Spain	F	3 y 10 m	Asymptomatic	Right medial UL	Spindle cell	G12S (34G>A)	S, ChT	Tumor-free age of 5 years and 4 months

AAC, amino acid change; ChT, chemotherapy; F, female; M, male; NDF, no data found; NS, nucleotide substitution; PT, patient; RT, radiotherapy; S, surgery; UL, umbilical ligament.  ^*∗*^Personal communication by author.

**Table 2:** Chemotherapy of patients with Costello syndrome and rhabdomyosarcoma.

PT number	Chemotherapy protocol
1	Initially with VCR, ACD, and CTX. After debulking continued on ChT using IFOS, VCR, and ACD. After the second course, when she developed IFOS induced encephalopathy, she was changed back to pulses of VCR, ACD, and CTX given every 3 weeks for 9 courses [7]
2	Initially IFOS, ACD, and VCR. After 4 courses, CTX was substituted for IFOS because hemorrhagic cystitis. Tx complicated by recurrent episodes of moderately severe diarrhoea and febrile neutropenia requiring readmission to hospital. Tx was completed after six courses of ChT [7]
3	Tx consisted of below the knee amputation and ChT (DOX, ACD, VCR, and CTX) [10]
4	Tx was implemented according to the Italian protocol for pediatric soft tissue sarcoma. Three courses of ChT, including VCR (0.025 mg/kg day 1), IFOS (50 mg/kg days 1 and 2), and alternating ACD (0.025 mg/kg day 1) and DOX (0.7 mg/kg day 1) were administered. Symptoms regressed, and the CT scan showed a reduction in tumor volume of more than two-thirds. After 3 more courses of ChT, the patient experienced abdominal pain. A repeat abdominal CT scan showed an increase in the pelvic mass. Despite the administration of a different ChT with CBDCA (11 mg/kg day 1) and VP-16 (3 mg/kg for 3 doses), the tumor continued to grow, and the child died 7 months after the diagnosis of RMS [12]
5	NDF [13]
6	The first cycle of ChT with VCR, ACD, and CTX and topotecan reduced the tumor size [16]. The tumor was surgically removed after the first ChT [8]. Debulking S was required because of rapid growth and tumor necrosis. The protocol was continued and was deemed sufficiently successful to withhold RT. One month after completion of the first protocol, tumor recurrence necessitated a second round of ChT with irinotecan and DOX. The latter drug was administered about 1 week before he died, during which time he had tachycardia (heart rate 180–20 bpm) [14]
7	The tumor did not respond to ChT and she died shortly after her 4th birthday [8]
8	Tx was a combination of S, ChT, and RT, continued until age of 3.5 years [8]
9-10	NDF [8]
11	PT had later generalized tonic-clonic seizures during her Tx for RMS [17]
12	Despite extensive ChT, the tumor progressed rapidly and the PT died 2 months later [18]
13	Induction ChT was commenced with the initial 12-week course of VCR, ACD, and CTX based on the Intermediate Risk Protocol D9803 of the COG [19]
14	ChT did not result in tumor shrinkage; therefore, surgical resection was performed [20]
15–22	NDF [5, 6, 9, 21–23]
23	ChT according to the cooperative soft tissue sarcoma protocol (CWS-2002 P) was well tolerated by the PT [24]
24	NDF [25]
25	ChT in the standard risk group protocol EpSSG RMS 2005 subgroup D, 3 preoperative cycles of IFOS (2 doses at 100 mg/kg), VCR (0.05 mg/kg), and ACD (0.05 mg/kg)
26	ChT (postoperative 15 days) in the standard risk group protocol EpSSG RMS 2005 subgroup B (Stage I), cycles of IFOS (2 doses of 3 g/m^2^/d), VCR (1.5 mg/m^2^), and ACD (1.5 mg/m^2^), was initiated

ACD, actinomycin D; CBDCA, carboplatin; ChT, chemotherapy; COG, children's oncology group; CTX, cyclophosphamide; CT, computed tomography; CWS, Cooperative Weichteilsarkom Studie (Cooperative Soft Tissue Sarcoma Study); DOX, Doxorubicin; EpSSG, European pediatric Soft tissue sarcoma Study Group; IFOS, ifosfamide; NDF, no data found; PT, patient; RMS, rhabdomyosarcoma; RT, radiotherapy; S, surgery; Tx, treatment; VCR, vincristine; VP-16, etoposide.

**Table 3:** Characteristics of Pediatric Patients with Umbilical Ligament Rhabdomyosarcoma.

PT number	Author	Year	Country	Sex	Age at diagnosis (years/months)	Symptoms	Location	Size (cm)	Histology	IRS group	Therapy	Outcome
1	Ransom [26, 27]	1933	USA	F	4 m	Mass below the umbilicus and extending somewhat to the right	Urachus (median UL): attached along the anterior wall from the umbilicus to the apex of the bladder	11.5	Spindle cell	I	SMAC, RT	Last check-up examination (2 years) showed no evidence of recurrence
2	Ishikawa et al. [28]	1985	Japan	F	1 y 4 m	Abdominal mass, abdominal pain, hemoperitoneum	Urachal (median UL): intra-abdominal rupture of the tumor	Fist-sized	NDF	IV	SMAC	NDF
3	Yokoyama et al. [27, 29]	1997	Japan	M	2 y	Episodic lower abdominal pain	Urachus (median UL): extraperitoneal located between the umbilicus and apex of the bladder and was adhering tightly to both. The bilateral medial UL were seen entering the tumor. Ruptured with a small number of disseminated tumors scattered on the mesentery and Douglas pouch	12	Embryonal	IV	SMAI, ChT	At 2 years of follow-up no recurrence had been detected
4^*∗*^	Kerr et al. [7, 8]	1998	England	F	3 y 2 m	Abdominal mass	Urachus (median UL), adherent to the bladder	NDF	Embryonal	IIa	SMII, ChT	Histology showed extending up to the resection margin. Died aged 5 years [5, 9]
5	Schulz and O'Leary [27, 30]	2001	Scotland	M	2 y	NDF	Urachus (median UL)	NDF	Embryonal	NDF	S, ChT, RT	Tumor-free age of 26 years
6	Fernández et al. [27]	2007	Spain	F	6 y	Intractable constipation, nausea, abdominal painless mass	Urachus (median UL), infiltrating the ventral abdominal wall and fixed to the vesical dome	13.5	Embryonal	Ib	SMIC, ChT	At 4 years of follow-up, the patient remains well and free of clinical disease
7^*∗*^	Menke et al. [24, 25]	2015	Germany	F	3 y	Abdominal mass	Lower anterior abdomen, above the urinary bladder^&^ (urachus, median UL by location)	NDF	Embryonal	IIa	SMII, ChT	R1 resection (marginal tumor residue). Tumor-free age of 6 years
8	Cheikhelard et al. [31]	2015 Dx: 1983	France	F	5 y 5 m	NDF	Urachus (median UL), peritoneal metastases and hypogastric lymph nodes	5	NDF	IV	SMAI, ChT	Died 31 months after diagnosis with relapse in peritoneal liver at 11 months
9	Cheikhelard et al. [31]	2015 Dx: 2003	France	F	3 y 3 m	Peritoneal rupture, poor general status	Urachus (median UL), lumboaortic and external iliac lymph nodes	10	Alveolar	IV	SMAI, ChT, RT	Died 57 months after diagnosis with relapse in peritoneal liver at 30 months
10	Cheikhelard et al. [31]	2015 Dx: 2005	France	F	2 y 6 m	Mass, poor general status, dysuria	Urachus (median UL)	15	Embryonal	III	SMII, ChT, RT	Died 37 months after diagnosis with peritoneal relapse at 19 months
11	Cheikhelard et al. [31]	2015 Dx: 2005	France	M	4 y 2 m	Mass, poor general status, dysuria	Urachus (median UL), peritoneal metastases and iliac lumboaortic lymph nodes	10	Embryonal	IV	SMII, ChT, RT	Relapsed multifocal at 82 months, follow-up 100 months
12	Cheikhelard et al. [31]	2015 Dx: 2005	France	M	5 y 3 m	Mass, poor general status	Urachus (median UL), peritoneal metastases and mesenteric lymph nodes	10.6	Embryonal	IV	SMIC, ChT, RT	Tumor free after 95 months since diagnosis
13	Cheikhelard et al. [31]	2015 Dx: 2006	France	M	2 y 5 m	Mass, poor general status, obstructive renal insufficiency	Urachus (median UL), peritoneal bone metastases and hypogastric lymph nodes	21	Embryonal	IV	SMII, ChT, RT	Bone progression after 16 months; died 18 after diagnosis
14	Cheikhelard et al. [31]	2015 Dx: 2010	France	M	4 y 5 m	Abdominal pain	Urachus (median UL)	7	Embryonal	III	SMIC, ChT	Tumor free after 18 months since diagnosis
15	Cheikhelard et al. [31]	2015 Dx: 2010	France	M	6 y	Abdominal pain	Urachus (median UL), peritoneal metastases	14	Embryonal	IV	SMAI, ChT, RT	Tumor free after 45 months since diagnosis
16^*∗*^	Sánchez-Montenegro et al. (this report)	2017 Dx: 2015	Spain	F	2 y 4 m	Asymptomatic	Left medial UL	5	Embryonal	Ia	ChT, SMIC, RT	Tumor-free age of 3 years and 9 months
17^*∗*^	Sánchez-Montenegro et al. (this report)	2017 Dx: 2015	Spain	F	3 y 10 m	Asymptomatic	Right medial UL	8.5	Spindle cell	IIa	SMII, ChT	Tumor-free age of 4 years and 5 months

ChT, chemotherapy; Dx, diagnosed; F, female; IRS, intergroup rhabdomyosarcoma; M, male; NDF, no data found; RT, radiotherapy; S, surgery; SMAI, surgery macroscopically incomplete; SMAC, surgery macroscopically complete; SMIC, surgery microscopically complete; SMII, surgery microscopically incomplete; UL, umbilical ligament.  ^&^Personal communication by author.  ^*∗*^Patients with Costello syndrome.

**Table 4:** Chemotherapy of pediatric patients with umbilical ligament rhabdomyosarcoma.

PT number	Chemotherapy protocol
1-2	No ChT [26, 28]
3	After establishment of the final diagnosis, he was treated with combined ChT, including VCR, ACD, and CTX [29]
4^*∗*^	Initially IFOS, ACD, and VCR. After 4 courses, CTX was substituted for IFOS because of hemorrhagic cystitis. Tx complicated by recurrent episodes of moderately severe diarrhoea and febrile neutropenia requiring readmission to hospital. Tx was completed after six courses of ChT [7]
5	NDF [30]
6	PT started induction of ChT 2 weeks after S. Induction regimen included 9 cycles of IVA (IFOS, VCR, and ACD) that were well tolerated with minimal systemic complications (Tx 953 of the MMT 95 SIOP protocol for RMS) [27]
7^*∗*^	ChT according to the cooperative soft tissue sarcoma protocol (CWS-2002 P) was well tolerated by the PT [24]
8	Adjuvant ChT with 10 cycles of IFOS, VCR, and ACD [31]
9	Adjuvant ChT with 6 cycles of IFOS, VCR, and VP-16 and 9 cycles of VCR, ACD, CTX/ACD, and CTX [31]
10	Neoadjuvant ChT with 6 cycles of IFOS, VCR, and ACD and adjuvant ChT with 3 cycles of IFOS, VCR, and ACD [31]
11	Neoadjuvant ChT with 1 cycle of CTX, VCR, and PRED, 2 cycles IFOS, VCR, and ACD, and 4 cycles of IFOS, VCR, ACD, and DOX and adjuvant ChT with 3 cycles of IFOS, VCR, and ACD [31]
12	Neoadjuvant ChT with 1 cycle of CTX, VCR, and PRED and 5 cycles of IFOS, VCR, ACD, and DOX and adjuvant ChT with 2 cycles of IFOS, VCR, ACD, and DOX and 1 cycle of IFOS, VCR, and ACD [31]
13	Neoadjuvant ChT with 2 cycles of VCR, ACD, and CTX + and 3 cycles of IFOS, VCR, ACD, and DOX and adjuvant ChT with 3 cycles of IFOS, VCR, and ACD [31]
14	Neoadjuvant ChT with 4 cycles of IFOS, VCR, ACD, and DOX and adjuvant ChT with 5 cycles of IFOS, VCR, ACD [31]
15	Neoadjuvant ChT with 4 cycles of IFOS, VCR, ACD, DOX, 3 cycles of IFOS, VCR, ACD and adjuvant ChT with 1 cycle of IFOS, VCR, ACD, 1 cycle of IFOS, VCR [31]
16^*∗*^	ChT in the standard risk group protocol EpSSG RMS 2005 subgroup D, 3 preoperative cycles of IFOS (2 doses at 100 mg/kg), VCR (0.05 mg/kg) and ACD (0.05 mg/kg)
17^*∗*^	ChT (postoperative 15 days) in the standard risk group protocol EpSSG RMS 2005 subgroup B (Stage I), cycles of IFOS (2 doses of 3 g/m^2^/d), VCR (1.5 mg/m^2^) and ACD (1.5 mg/m^2^) was initiated

ACD, actinomycin D; ChT, chemotherapy; CTX, cyclophosphamide; CWS, Cooperative Weichteilsarkom Studie (Cooperative Soft Tissue Sarcoma Study); DOX, Doxorubicin; EpSSG, European pediatric Soft tissue sarcoma Study Group; IFOS, ifosfamide; MMT, malignant mesenchymal tumor; NDF, no data found; PRED, prednisone; PT, patient; RMS, rhabdomyosarcoma; S, surgery; SIOP, Société Internationale D'Oncologie Pédiatrique (International Society of Pediatric Oncology); Tx, treatment; VCR, vincristine; VP-16, etoposide.  ^*∗*^Patients with Costello syndrome.
